# Commercial microbiota test revealed differences in the composition of intestinal microorganisms between children with autism spectrum disorders and neurotypical peers

**DOI:** 10.1038/s41598-021-03794-8

**Published:** 2021-12-20

**Authors:** Magdalena Jendraszak, Mirosława Gałęcka, Małgorzata Kotwicka, Aleksandra Regdos, Michalina Pazgrat-Patan, Mirosław Andrusiewicz

**Affiliations:** 1 Chair and Department of Cell Biology, Faculty of Health Sciences, Poznan University of Medical Sciences, Rokietnicka 5D, 60-806 Poznan, Poland; 2 Institute of Microecology, Sielska 6, 60-129 Poznan, Poland

**Keywords:** Microbiology techniques, Applied microbiology, Gastroenterology, Autism spectrum disorders

## Abstract

The early-life modifications of intestinal microbiota may impact children's subsequent emotional and cognitive development. Studies show that some bacteria species in gut microbiota, and the lack of others, may play a key role in autism spectrum disorders (ASD) development. Fecal samples were obtained from three groups of children: 16 healthy, 24 with allergies (ALG), and 33 with ASD (probiotics and non-probiotics users). The analysis was carried out according to the KyberKompakt Pro protocol. We observed a significantly higher level of *Klebsiella* spp. in the healthy children from the non-probiotics group, considering three groups. In the same group, *Bifidobacterium* spp*.* the level was lower in ASD compared to neurotypical individuals. In healthy children who did not use probiotics, strong positive correlations were observed in *E. coli* and *Enterococcus* spp*.* and *Bacteroides* and *Klebsiella* spp., and a negative correlation for *Akkermansia muciniphila* with both *Klebsiella* spp*.* and *Bacteroides* spp*.* In the ASD group who take probiotics, a strongly negative correlation was observed in *Lactobacillus* spp., and both *Faecalibacterium prausnitzii* and *Akkermansia muciniphila* levels*.* In the ALG group, the strongest, negative correlation was found between *Enterococcus* spp*.* and *Lactobacillus* spp*.* as in *Akkermansia muciniphila* and *Bifidobacterium* spp. The simple commercial test revealed minor differences in the composition of intestinal microorganisms between children with autism spectrum disorders and neurotypical peers.

## Introduction

The human gut microbiota is a complex, non-homogenous ecosystem represented by 10^13^–10^14^ microbes, with over a thousand different species, which possess a 100-fold more genes than found in the human genome. Strict anaerobic bacteria are the primary microcrobes found in the gut, but protozoa, fungi, archaea, and viruses are also detectable. Microbiological colonization of the intestines begins during childbirth. The type of delivery (vaginal or Caesarean section) and gestational age of birth (pre-term or full-term) may play a significant role in post-natal development, as well as in the maturation of endocrine, immune, and nervous systems^1–3^. According to recent studies, early-life modifications of intestinal microbiota may affect subsequent emotional and cognitive development. The diversity of gut microbiota may be crucial for the successful implementation of behavioral skills and proper brain development^4–6^.

In recent years, an increasing number of studies reported that gut microbiota might participate in the process of maintaining human homeostasis through the regulation of mood and well-being, involvement in enteric and central nervous system development, and controlling appetite and metabolism. A bidirectional communication pathway exists between intestinal microorganisms and the brain, known as the gut-brain axis, enabling gut microbes to communicate with the brain, while also acting in an inverse manner. The gut-brain communication mechanisms are complex and have not yet been completely defined. They are assumed to involve many different axes, including immune, neural, endocrine, and metabolic pathways. It is suggested that communication between gut microbiota and the brain occurs through the vagus nerve, the immune system, gut hormone signaling, or microbial metabolites, including short-chain fatty acids (SCFA) and neurotransmitter molecules. It has been demonstrated that gut microbiota might directly affect the brain by production of several bacterial molecules essential for brain functioning e.g.: tryptophan, serotonin, dopamine, kynurenine, and γ-aminobutyric acid^5–10^.

However, both clinical and experimental evidence have shown that the homeostatic disorder of gastrointestinal microbiota (dysbiosis) can be related to a wide range of various types of non-neurological diseases e.g.: type 2 diabetes, inflammatory bowel disease, allergies, necrotizing enterocolitis in infants, infections, and obesity^11–14^. On the other hand, dysbiosis can lead to neurological changes such as autism, schizophrenia or Parkinson's disease^5,15^ and can impact the severity of psychiatric disorders including depression, stress or anxiety^7,16,17^.

Autism spectrum disorders (ASD), referenced by neurological and developmental dysfunction, are manifested by deficiencies in social communication skills, lack of reciprocal social interactions, and unusual repetitive behaviors. Generally, ASD includes different developmental disorders: the classic form of autistic disorder, Asperger's Syndrome, and Pervasive Developmental Disorder—Not Otherwise Specified^18^. Various studies have shown that genetic implications and environmental factors (chemicals, drugs, diet, prenatal viral infections) can be associated with ASD etiopathogenesis^19–21^. Moreover, according to recent findings, the abundance of some bacterial species, as well as intestinal microbiota composition, may play a crucial role in ASD development and gastrointestinal (GI) problems, which is characteristic for individuals with autism and can be due to gut dysbiosis. A link between alterations in gut microbiota composition and ASD is not well established^22–27^.

Several studies have reported children with ASD present more frequently with gastrointestinal problems such as abdominal pain, constipation or diarrhea, bloating, and/or gastroesophageal reflux than in healthy individuals. In turn, chronic Gl disturbances may also aggravate behavioral problems, such as frustration and aggression, and are speculated to correlate with the severity of autism^28–32^. The cause of these intestinal disorders is unknown. However, they appear to be associated with a disarrangement of gut microbiota, particularly, in the excessive growth of pathogenic bacteria (e.g. *Clostridium* spp.) and the decrease of beneficial microorganisms (such as *Lactobacillus* and *Bifidobacterium*)^27,33,34^. Many studies have demonstrated that the fecal microbiota of autistic children differs significantly from the fecal microbiota of neurotypical children. These results are often inconsistent and are not supported by clinical trials based on a large patient group. Moreover, various reports show that the content of the same species of bacteria may be higher or lower in people with ASD. For example, in research carried out by De Angelis et al., the level of *Lactobacillus* spp*.*, as well as the *Bacteroidetes/Firmicutes* ratio, in fecal samples of children with autism was lower compared to healthy participants while *Clostridium* spp. was overrepresented^27,33^. In contrast, Williams et al. noticed an increased *Bacteroidetes/Firmicutes* ratio in ASD samples^35^. Conversely, Tomova et al. showed a significant decrease in *Bacteroidetes/Firmicutes* ratio but an increase in *Lactobacillus* spp. and *Clostridium* spp. in autistic children^29^. Other microbes observed in large quantities in the feces of ASD compared to neurotypical children include such species as *Akkermansia muciniphila* and *Prevotella*^33^ or *Desulfovibrio*^36^, is contrary to the lower or unchanged level of *Akkermansia muciniphila*^37,38^, and *Prevotella* and *Desulfovibrio*^38^*.*

The purpose of this study was to examine selected gut microorganisms, both beneficial and pathogenic, in the feces of three groups of children: healthy, with allergies (ALG), and with ASD. Children with allergies were treated as the positive control group. Multiple studies have reported that gut microbiota of allergic patients shows, similar to ASD children, a significant abnormalities in the composition of gut microorganism^39–41^. In our study, we evaluated 19 gut microorganisms by comparing their composition in ASD subjects to neurotypical children. However, our study has some limitations: small sample size, limited precision of the methods, and a limited number of bacteria. We also considered differences and similarities between groups, trying to determine if microbiota imbalances could be the basis for manifestation of, or a marker for, ASD. Our analysis based on the diagnostic intestinal microbiota test enables the detection and identification of foundation and keystone bacterial species in the intestinal ecosystem. Nowadays, diagnostic tests for gut microorganisms are becoming easily available and increasingly used in commercial diagnostics. In this way, we tried to emphasize the practical application of scientific research and link it with the diagnostic process.

## Results

**Figure 1:**
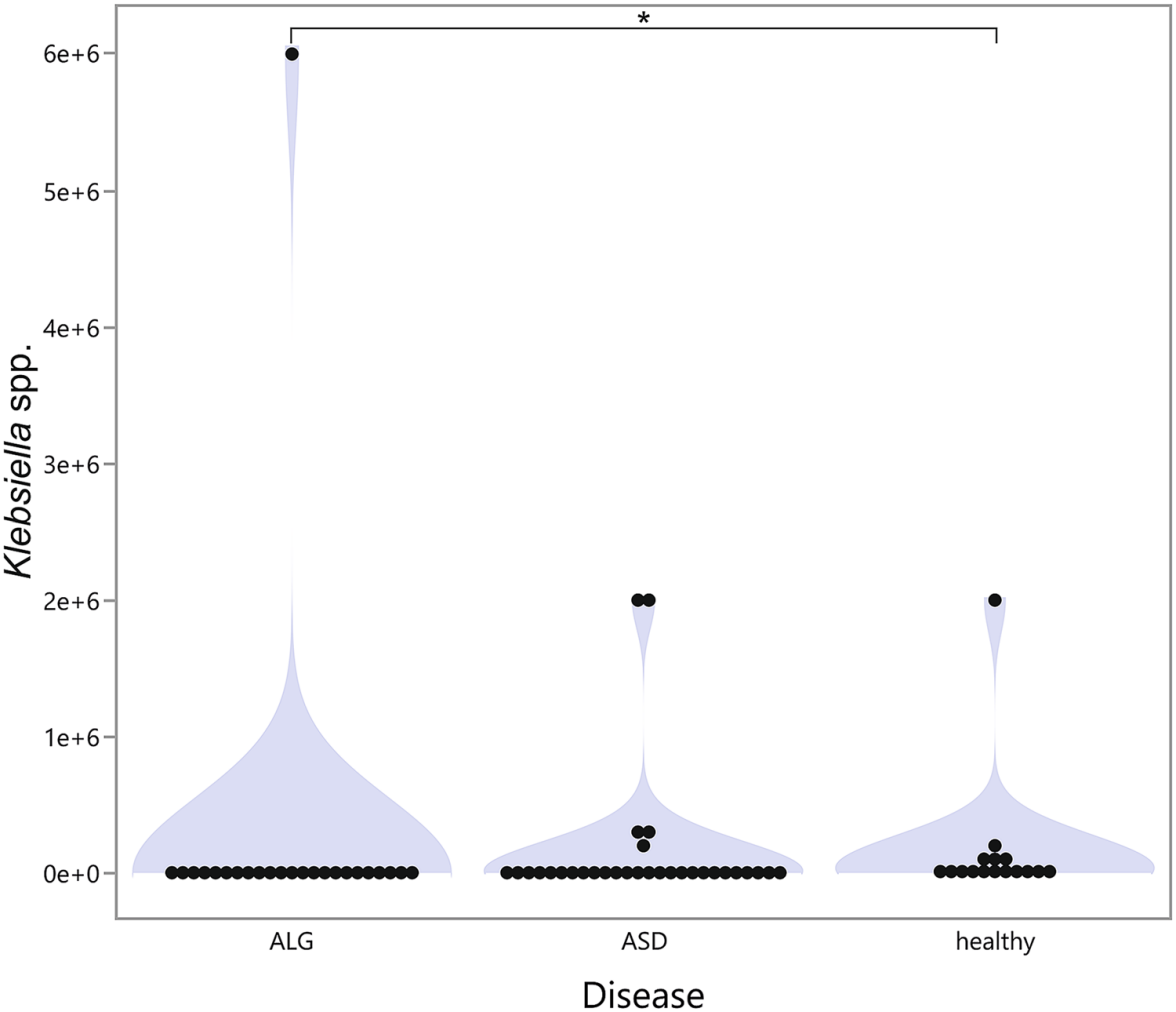
Violin plot of *Klebsiella* spp*.* level in the stool of children with ASD, allergies, and in the healthy group regardless of probiotic usage. *p < 0.05.

Considering all three analyzed groups, we observed a significantly higher level of *Klebsiella* spp. in the healthy group (*p* = 0.0055), regardless of probiotic usage. The Dunn *post-hoc* test showed a significantly higher median level of *Klebsiella* spp*.* in the healthy group compared to ALG children (*p* = 0.0199), with a wider range in children with allergies, Fig. 1. No other differences were found regardless of the bacteria species.

**Figure 2:**
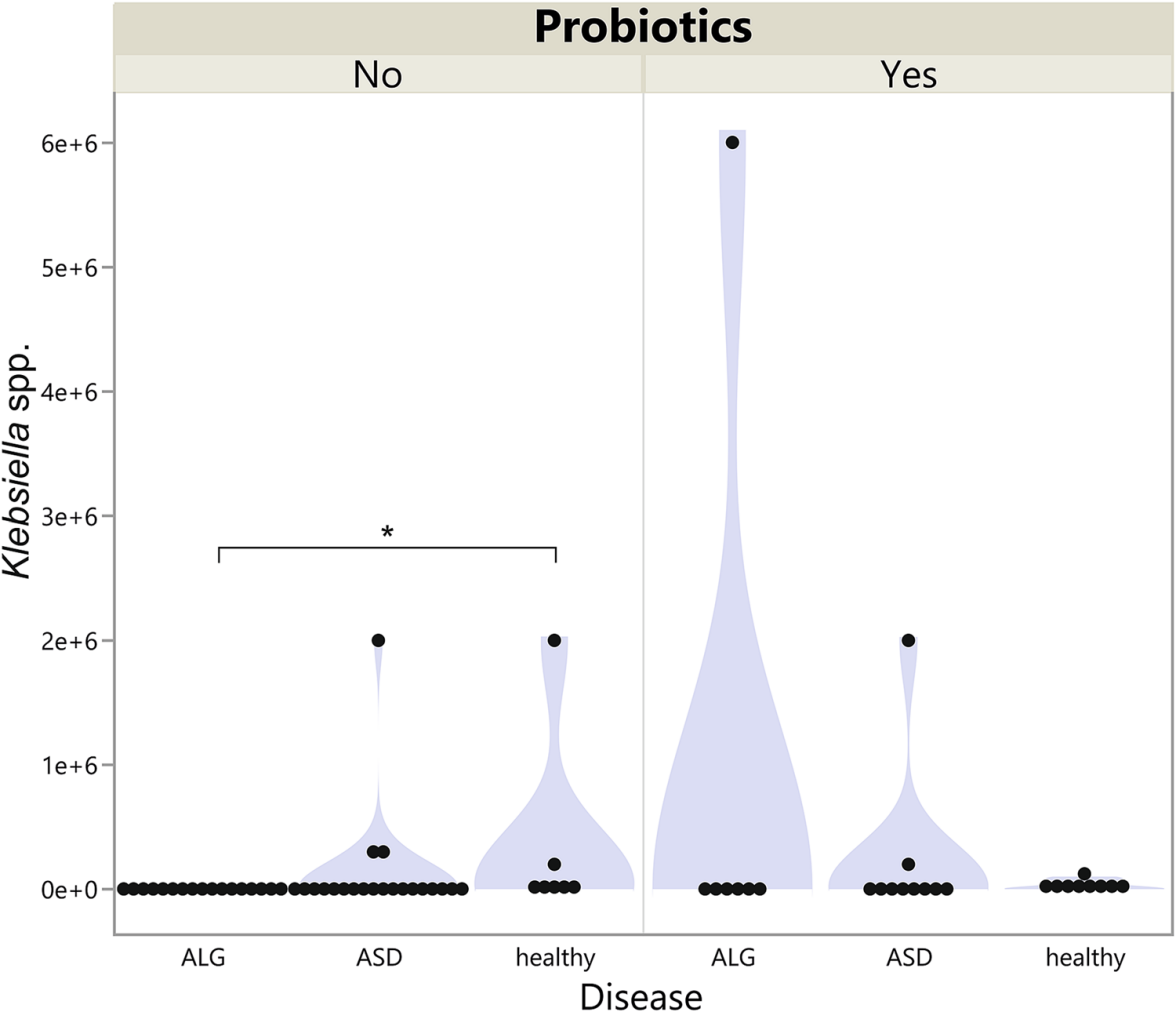
Violin plot of *Klebsiella* spp*.* level in the stool of children with ASD, allergies, and in the healthy group divided by probiotic usage. *p < 0.05.

Taking into consideration probiotics usage, a significant difference in the *Klebsiella* spp. level (*p* = 0.004) was observed in the non-probiotics administrated group with the highest level in healthy children. The Dunn *post-hoc* test showed that the levels differed between the healthy and ALG groups (*p* = 0.028). In children who used probiotics, the difference was not significant (*p* = 0.593, Fig. 2). The range was highest in the ALG group, but the median value was slightly higher in the healthy control.

**Figure 3:**
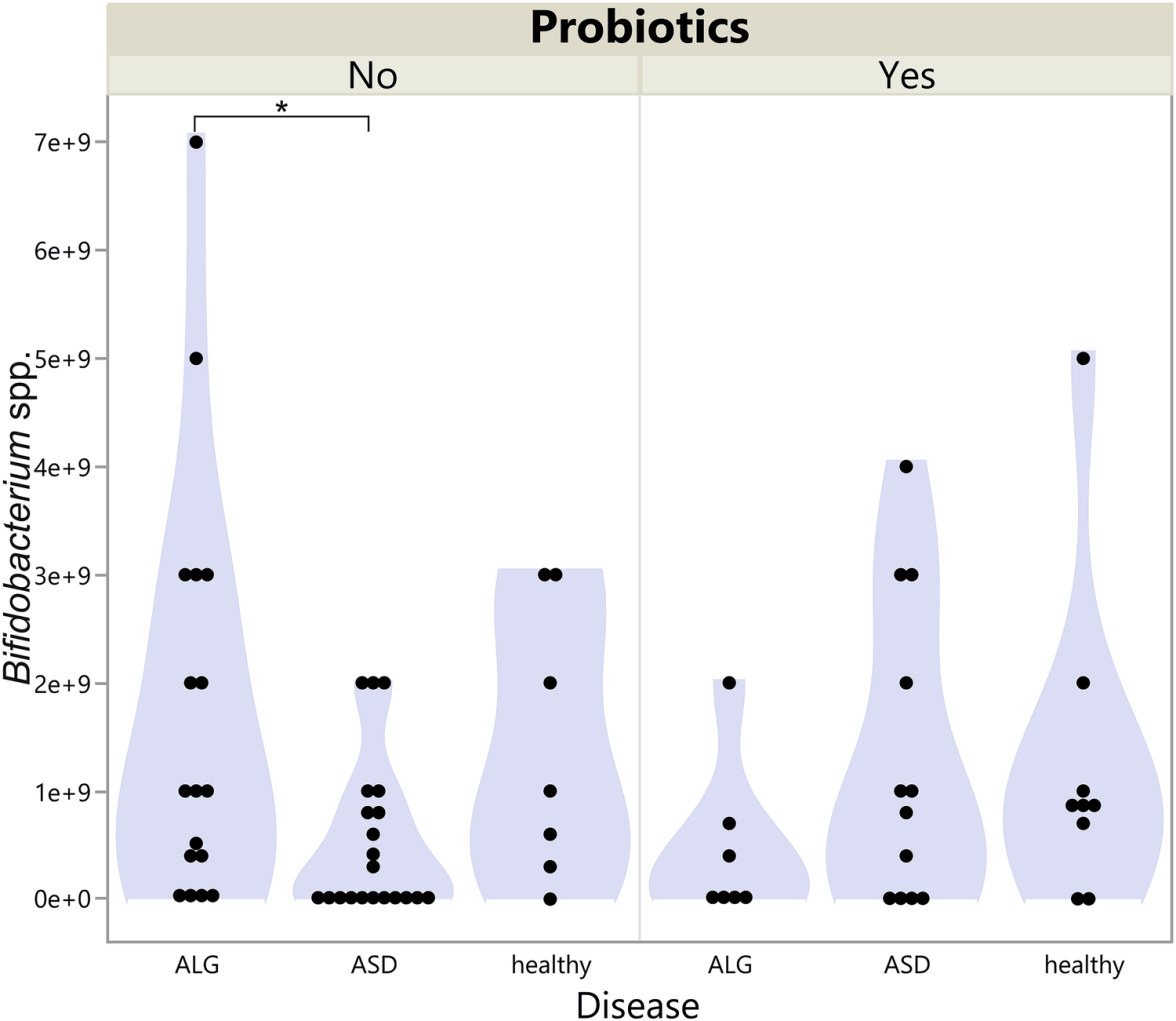
Violin plot of *Bifidobacterium* spp*.* level in the stool of children with ASD, allergies, and in the healthy group divided by probiotic usage. *p < 0.05.

When probiotics were not administered, there was a significant difference in *Bifidobacterium* spp. (*p* = 0.029) in the ASD and ALG group (*p* = 0.036) as determined by the Dunn *post-hoc* test. The highest level was observed in the ALG children. When probiotics were used, the difference was not significant (*p* = 0.278, Fig. 3). The range and median were highest in the healthy controls.

**Figure 4:**
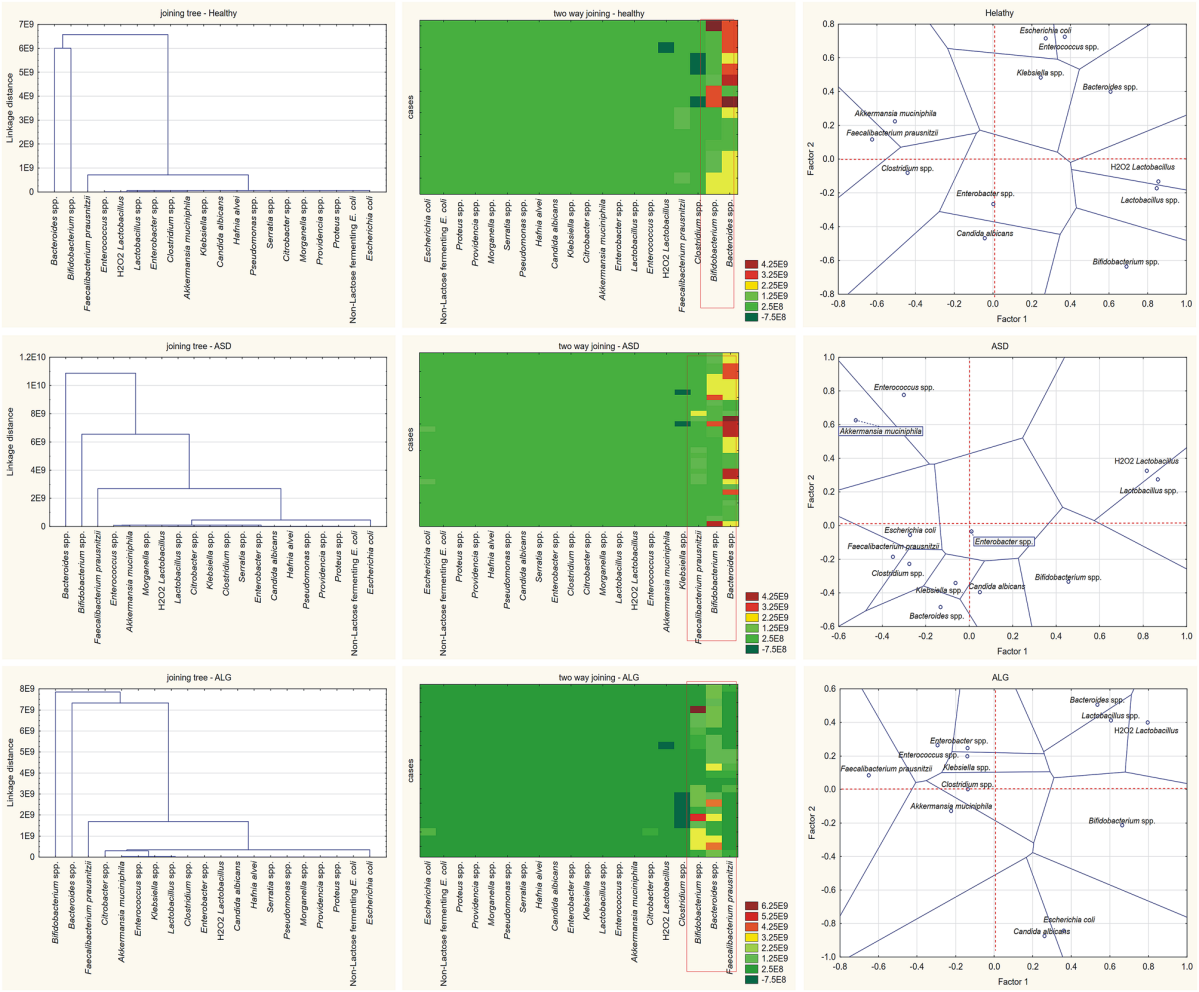
Agglomeration analysis using Euclidian distances, to way joining and correlation matrix for factor analysis of principal components loadings for multi-presence multiple-bacteria species.

Agglomeration analysis using Euclidian distances (single linkage analysis) Ward's method, was computed for bacteria in the stool. Additionally, the correlation matrix for factor analysis of principal component loadings for multi-presence, multiple-bacteria species with unrotated factor rotation was computed to obtain homogenous subgroups. As presented in Fig. 4, differences in bacteria species presence, distribution, and coexistence in ASD, ALG and healthy group is shown. Although the joining tree shows similar coexistence of different species of bacteria, however, the two way-clustering differs between the health status in some cases. It is especially evident in the variation level of bacteria marked with red boxes and could point dysbiosis. *Bifidobacteria* and *Bacteroides* vary the most, but as shown in Fig. 4, in the ASD and the ALG children's stool, the *Faecalibacterium prausnitzii* level was highly variable. Factor analysis of principal component loadings for multi-presence, multiple-bacteria species highlights the changes in bacterial coexistence. Some species, due to lack of variation in the subgroups, were excluded from the analysis. The adjacent species and the Voronoi tessellation lines show the different presence of the bacteria in the stool and define adjacent polygons, including all peaks in the output plot. As expected, *Lactobacillus* spp*.* and H_2_O_2_ *Lactobacillus* were in a similar distance, always in the same quadrant and near *Bacteroides*. Surprisingly, *E. coli* and *C. albicans* were near in the ALG and ASD group but not in healthy children. *Akkermansia muciniphila* was distant from *Bacteroides* in all analyzed groups. It was not possible to perform the analysis based on probiotic usage due to an insufficient number of cases in those divisions.

Descriptive statistics as a summary of various microorganism species' concentration investigated in the stool of children were shown in Supplementary materials (Tables S1, S2, and S3). The Supplementary data show values in the whole group, health status divided, and probiotic usage categorized. The *Clostridium difficile* and molds, due to a lack of conclusive data for all cases, were excluded from statistical analysis.

In the non-probiotic, healthy control group, the Spearman's rank correlation showed a significant, positive, and strong correlation between *E. coli* and *Enterococcus* spp*.* and *Bacteroides* and *Klebsiella* spp*.* (both, R = 0.79). A negative correlation was observed in *Akkermansia muciphila* with both *Klebsiella* spp*.* and *Bacteroides* spp*.* (R =  − 0.82 and R =  − 0.80, respectively). In healthy children that used probiotics, a significant, strong, and positive correlation was shown in the case of the *Bacteroides* spp*.* level *vs. Bifidobacterium* spp. and *Akkermansia muciniphila vs. Faecalibacterium prausnitzii* (both, R = 0.68), and a strong negative correlation of *Akkermansia muciniphila* level *vs. C. albicans* (R =  − 0.7).

In the ASD group without probiotics, a moderately positive correlation was observed in *Lactobacillus* spp*.* level *vs. Bifidobacterium* spp*.* (R = 0.58) and *C. albicans* (R = 0.44). Negative correlations were observed in *Akkermansia muciniphila vs*. both *Bifidobacterium* spp. and *Lactobacillus* spp*.* (R =  − 0.44 and R =  − 0.49, respectively). *C. albicans* negatively correlated with *Faecalibacterium prausnitzii* (R =  − 0.47). In the ASD group taking probiotics, a strongly negative correlation was observed in the case of *Lactobacillus* spp*. vs.* both *Faecalibacterium prausnitzii* and *Akkermansia muciniphila* (R =  − 0.78 and R =  − 0.64, respectively). Additionally, the level *Akkermansia muciniphila* positively correlated with *Faecalibacterium prausnitzii* (R = 0.59).

Assessment of the ALG group who did not take probiotics showed a significant, moderate, and positive correlation in the case of *Bacteroides* spp. and *Lactobacillus* spp. (R = 0.48) as well as with *C. albicans vs*. both *Citrobacter* spp. and *Clostridium* spp*.* (R = 0.49 and R = 0.63, respectively). A negative correlation was observed in the case of *Faecalibacterium prausnitzii vs. Lactobacillus* (R =  − 0.53). In the ALG group with probiotics, *Enterococcus* spp. positively correlated with *Lactobacillus* spp. (R = 0.90), and a negative correlation was observed in both *Akkermansia muciniphila vs*. *Bifidobacterium* spp. and *Bacteroides* spp*.* vs. *C. albicans* (R =  − 0.81 and R =  − 0.79, respectively).

## Discussion

Increasing evidence suggests the balance and diversity within the bacterial population are essential in maintaining proper function of the gastrointestinal tract and immune system as well as human homeostasis. On the other hand, there are a wide range of indicators that propose an imbalance of the gut microbial ecosystem may lead to inflammation and immune activation in several disorders such as gastrointestinal diseases, cardiovascular disease, metabolic and psychiatric disorders, allergy, or asthma^5,11,14,15,40^.

The pathogenesis of ASD is complex, and apart from genetic factors, environmental factors such as the intestinal community, may play a key role in the symptomology of ASD. The composition of gut microorganisms that increase susceptibility to autism development, as well as evidence linking autism symptoms and intestinal dysbiosis, have yet to be fully explained^18–20,24,42^. However, frequent occurrence of GI symptoms in ASD children suggest the involvement of the gut microbiota in gastrointestinal pathophysiology which then constitute potential diagnostic and therapeutic targets. It was suggested that dietary intervention (gluten-, casein-, and soy-free diet), probiotic/prebiotic treatment, microbiota transfer therapy, or targeted antibiotic therapy could be a new strategy for treatment. It could help children with chronic gastrointestinal disorders and may reduce ASD symptoms by improving language, cognitive skills, and behavioral deficits^43–46^.

Our results showed minor differences in the composition of intestinal microorganisms between children with autism spectrum disorders and neurotypical individuals. In non-probiotics cases, considering three groups, we observed a significantly higher level of *Klebsiella* spp. in the healthy children. In the same group, *Bifidobacterium* spp*.* the level was lower in ASD and the highest in ALG group.

Recent studies examining the association of microbiota and children with autism suggest excessive use of antibiotics in ASD individuals may cause an overgrowth of certain *Clostridum* species such as *C. tetani*, *C. perfringens,* or *C. difficile.* According to the hypothesis linking the occurrence of *Clostridium* with the etiopathogenesis of autism, an overgrowth of some toxin-producing *Clostridum* species can expose ASD children to high levels of microbial neurotoxic metabolites. This thereby affects normal nervous system development and leads to the exacerbation of gastrointestinal problems^26,34,47–49^.

Moreover, the anaerobic bacteria *Clostridium* and *Bacteroides* are sources of short-chain fatty acids (SCFA), such as propionic, acetic, butyric, and valeric acid, usually produced during fiber fermentation. These metabolites are believed to be involved in gut immune system function, modulation of the nervous system through the gut-brain axis, and host cell gene expression^25,33,50^. SCFAs can induce widespread effects on the human organism, but an imbalance in their levels may change intestinal homeostasis and cause peripheral inflammation. SCFAs reach the brain through blood circulation and affect its development by modulating production of serotonin and dopamine. High concentrations of propionic acid, a significant neurotoxic metabolite, may disrupt brain function, resulting in developmental delay or regression^25,51–53^.

However, the presence of specific *Clostridium* species, clusters, and their content in the intestinal microbiome of ASD children is still under discussion. Moreover, current results are often inconclusive, and the contribution of selected species in ASD etiology have yet to be fully explained. Our analysis showed no significant differences in the levels of *Clostridium* spp. within the groups. The results, therefore, are similar to those of Wang and Iovene^37,54^ but are not consistent with other reports that found increased *Clostridium* in the stool of ASD children^33,49,55,56^.

*Bacteroides* spp. and *Clostridum* spp. are defined as bacteria associated with fiber fermentation and SCFA/propionic acid production. It has been suggested that neurodevelopmental disorders in ASD patients correlate with impaired propionic acid metabolism and changes in propionate producing bacteria^53,57^. In our analysis, the level of *Bacteroides* is unchanged in all analyzed groups. These findings are comparable with those of Parracho et al. and Ma et al.^55,58^ but contrasts other studies where increased *Bacteroides* in ASD patients has been reported^33,50^. Moreover, our analysis showed no significant changes in the abundance of *Enterobacteriaceae* family in stool samples of any studied groups, except a higher level of *Klebsiella* species in the healthy individuals. This result is compatible with Adams’ observation^28^. However, in the microbiome of the ASD group, a significant increase of *Proteobacteria* phylum, particularly species belonging to *Enterobacteriaceae,* was observed^33,50,59^.

Additionally, in healthy controls, a significantly positive and strong correlation of *Escherichia coli* and *Enterococcus* spp*.* was noted. It is well-known that certain strains of *E. coli* and *Enterococcus* have probiotic properties and can activate the gut mucosal immune system by increasing antibody quantities and cytokine production and also improve the barrier function of the intestinal epithelium^60–62^. Cukrowska et al. has reported the presence of probiotic *E. coli Nissle 1917* in infant's intestines may enhance the humoral immune system response, especially the induction of specific IgA and IgM antibodies^63^. Hafez has demonstrated that this beneficial strain may regulate mucin gene expression, thereby altering the intestinal mucus layer and indirectly regulating the gut immune system^64^.

Our results showed lower levels of *Bifidobacterium* in ASD, which is compatible with several other studies^28,33,37,50^. We speculate this may be due to a derangement of the probiotic bacteria population in the intestines of autistic children. Some studies have also indicated varying levels of probiotic bacteria such as *Lactobacillus* and *Bifidobacterium* in the intestines of ASD and neurotypical subjects^26,28,29,33,37,50^. On the other hand, similar numbers of *Bifidobacterium* in ALG and healthy groups may be a compensatory mechanism. Allergies are chronic inflammatory diseases, and this group of bacteria shows strong anti-inflammatory properties. Recently published studies have reported that *Bifidobacterium* strains may inhibit the inflammatory response and exert an immunomodulatory effect by stimulating IL-10 or IL-12 synthesis by dendritic cells^65,66^. Furthermore, the presence of both probiotic bacteria in the intestines contributes to maintenance of the epithelial barrier integrity and protects against an overgrowth of pathogens^54,67^. Additionally, they can impact the metabolism of toxins, drugs, and some dietary compounds as well as gut epithelial cell proliferation^67–69^. Interestingly, both genera may produce γ-aminobutyric acid (GABA), the primary inhibitory neurotransmitter in the brain^25,68^. According to some studies, lower GABA levels are correlated with anxiety and social disorders in ASD individuals^70,71^.

Moreover, some *Lactobacillus* and *Bifidobacterium* strains are the main components of probiotic supplements. Growing clinical evidence suggests the consumption of oral probiotics reduce GI discomfort, modulate the stress response, and improve mood and anxiety symptoms in patients with ASD^28,29,72,73^. However, in our analysis, we observed a strong negative correlation between probiotic bacteria and *Akkermansia muciniphila* and *Faecalibacterium prausnitzii* levels in ALG and ASD groups using probiotics. We assume this may be due to the dominant role of some probiotic strains or as a result of nutrient competition. Both *A. muciniphila* and *F. prausnitzii* are considered biomarkers of healthy intestinal flora and modulators of immune system^74,75^. Additionally, *Faecalibacterium* may regulate the expression of interferon-gamma (IFNγ), which plays an indirect role in neuroplasticity and synapse formation^25,76^. Based on these factors, it can be assumed in children with these associated diseases, selection of appropriate probiotic strains is important, and probiotic therapy should be performed on the basis of previous microbiota analysis.

In our studies, we estimated the content of fecal fungi, especially *Candida* genus, in ASD children. The healthy gut is colonized by yeast and good bacteria living in balance with one other. Most *Candid*a species are harmless commensals, but when intestinal homeostasis is disturbed, they can cause infections called candidiasis. Yeast infections have been rarely investigated in autistic individuals. Our studies have shown no significant differences between groups. However, some investigators report substantial growth of *Candida*, particularly *Candida albicans*, in ASD patients^26,54,77,78^, Contrary to these results, Adams et al. did not confirm these findings^28^. The potential role of the *Candida species* in ASD etiology is unclear, and further studies are needed. It is believed that an overgrowth of *Candida* spp*.* may induce autistic behavior through excessive production of ammonia which then is converted to beta-alanine, a non-essential amino acid structurally similar to the inhibitory neurotransmitter GABA^78,79^. Additionally, a high abundance of yeast may impair the absorption of both carbohydrates and mineral elements, as well as affect the release and accumulation of toxins^77,79^. Moreover, enormous growth of *Candida* in the gut of autistic individuals may aggravate GI abnormalities by dysregulation of cytokine release^26^.

It should be noted that our study has some limitations, e.g., heterogeneity between groups, small sample size, limited precision of methods, and limited number of bacteria. This fact could be the cause of not having found significant differences in the comparison between the microbiota profile. However, we would like to highlight that it was not our aim to detect bacterial species/clusters associated with the development of ASD. We wanted to show whether, using simple but supported by scientific research, diagnostic methods, we are able to find bacterial bioindicators distinguishing people with ASD from neurotypical. In turn, the similarities/differences between the groups could help select the appropriate probiotic and diet for the patients.

## Conclusions

The results of our study do not fully support the hypothesis that the composition of the gastrointestinal microbiota, the presence of certain species, or significantly altered ratios of these microbes change susceptibility to ASD development of children. The formation of intestinal microorganisms is influenced by many factors such as the type of delivery or feeding, child's diet later in life, and even geographical location. Typical research methods are often heterogeneous and do not include this additional information. However, it cannot be excluded that ASD etiopathogenesis is likely multifactorial and involves multiple etiopathogenic mechanisms. Despite the complexity of this issue, it can be assumed that the increased abundance of certain harmful bacterial species, as well as reduction of beneficial ones, in autistic individuals may result in intensified gastrointestinal problems. For these reasons, an analysis of intestinal microbiota along with an exclusion diet enriched with probiotic/prebiotic supplementation could help alleviate GI symptoms and improve the quality of life of ASD children. Additionally, we would like to point out that children were supplemented with probiotics in an uncontrolled manner, beyond a physician’s control. In the context of our results, it seems essential that before using probiotic therapy, children’s gut microbiota should be tested to supplement their diet with appropriate strains of probiotic bacteria. Moreover, it looks as that probiotic therapy, especially in children, should be carried out under specialist supervision.

## Materials and methods

### Participants

This study aimed to compare 89 children’s stool samples that were screened for selected gut microorganisms at the Institute of Microecology (Poznan). The children's parents completed a self-reporting questionnaire consisting of a set of questions regarding their child's: age, sex, body mass, height, health status, probiotic or/and antibiotic supplementation, and radiotherapy/chemotherapy treatment. The information included in the questionnaires was verified in a direct interview, by e-mail, or by telephone interview. The diagnosis of ASD was carried out in various psychological centers. In Poland, a two-stage procedure is used to diagnose autism in a child. The first stage (screening test) included: The Modified-Checklist for Autism in Toddlers (M-CHAT), or Quantitative Checklist for Autism in Toddlers (Q-CHAT), or The Childhood Autism Spectrum Test (CAST). The second stage included: Autism Diagnostic Interview-Revised (ADI-R), and Autism Observation Schedule – Second Edition (ADOS-2), and The Childhood Autism Rating Scale (CARS). We have created a group of allergy sufferers based on a self-report questionnaire about the kind of allergy. In the statistical analysis, we limited the children with allergies to those with food allergies and atopic dermatitis. According to their parents' declarations, children did not take any medications. Based on this information, three children's groups were distinguished: without existing illnesses, with allergies, and with ASD. The individuals with autoimmune diseases, Lyme disease, diabetes, cancer, and children who had undergone antibiotic therapy within 3 months, radiotherapy, and chemotherapy were excluded from further analysis. Neurotypical children and allergy sufferers were not related to any degree with ASD children. The children were enrolled regardless of any gastrointestinal disturbances. The self-report questionnaire consisted of gastrointestinal symptoms questions such as diarrhea, constipation, bloating, pancreatic diseases, liver disease, celiac diseases, or lactose intolerance. Patients reported problems with constipation and flatulence. Two children in the ASD group were additionally diagnosed with lactose intolerance. According to the exclusion criteria, 73 children were included in further statistical analysis: 16 healthy children, 24 with allergies, and 33 with ASD. Based on the parents’ answers in the questionnaire, we distinguished: in the ASD group, 9 out of 33 children (27%) and 7 out of 27 (29%) of the ALG group reported gastrointestinal symptoms, while none of the children in the healthy group. The descriptive statistics for the study group was shown in Table 1. The healthy children and those with allergic diseases were treated as negative and positive controls, respectively. The data were analyzed between the illness status groups as well as probiotic usage. Before the microbiota examination, the children took various probiotics containing mainly *Lactobacillus* and *Bifidobacterium* strains (Dicoflor 30, Vivomixx, Multilac, Sanprobi). The number of strains in the probiotics ranged from 2 to 4, and the number of bacteria ranged from 3 to 10 × 10^9^. The duration of probiotic supplementation was heterogeneous and ranged from 3 months to 2 weeks. However, to create a homogeneous group, we limited children who took probiotics between 3 and 1 month. The cut-off point was 4 weeks of taking the probiotic because, according to some reports, such time could affect the gut microbiota. The descriptive statistics for the study group was shown in Table 2. Informed consent was obtained from all parents or legally authorized representatives, and identifying information was removed from each sample. The study protocol was in accordance with the Declaration of Helsinki and was approved by the Ethical Committee of Poznan University of Medical Sciences. However, it concluded that it is not a clinical trial and does not have the characteristics of a medical experiment (date of approval: 08/12/2020). According to the regulations of local IRB our study requires fully anonymized data of the participants. All microbiological diagnostic tests were performed under the supervision of a diagnostician at the Institute of Microecology in Poznan.

**Table 1:** Summary of subject characteristics.

	Healthy	ASD	ALG
Total participants^a^	16 (22%)	33 (45%)	24 (33%)
Female/Male^a^	6 (38%)/10 (63%)	4 (12%)/29 (88%)	9 (38%)/15 (62%)
Age (year)^b^	5.5 [3–9]	5 [4–6]	7 [4.5–9.5]
BMI^b^	14.88 [14.20–16.97]	14.86 [14.06–16.02]	15.16 [14.18–18.07]
Probiotic usage [yes/total]^a^	9/16 (56%)	12/33 (36%)	7/24 (29%)
Gastrointestinal disorders^a^	0	9 (27%)	7 (29%)

*ALG* allergies, *ASD* autism spectrum disorders, *BMI* body mass index.

^a^Number of cases (percentage).

^b^Median [interquartile range].

**Table 2:** The age of children according to probiotics supplementation.

	Probiotics using	Non-probiotics
Total participants^a^	28 (38%)	45 (62%)
Female/Male^a^	9 (12%)/19 (26%)	10 (14%)/35 (48%)
Age (year)^b^	5 [4–8]	5 [3–7]
BMI^b^	14.99 [14.12–16.97]	14.88 [14.06–16.66]
Gastrointestinal disorders^a^	9 (32%)	7 (15%)

*BMI* body mass index.

^a^Number of cases (percentage).

^b^Median [interquartile range].

## Materials and procedures

### Collection and preparation of stool samples

Stool samples were collected in sterile stool tubes at the participants' homes and delivered to the Institute of Microecology (Poznan), where they were analyzed for selected intestinal microorganisms. The analysis was carried out following the KyberKompakt Pro protocol and included both microbiological cultures and quantitative polymerase chain reactions (qPCR). All counts were expressed as the numbers of log_10_ CFU (colony-forming unit) per 1 g of sample.

### Microbiological identification of selected microorganisms

Before microbial culture, 0.25 g of each sample was diluted ten times in 0.85% sterile NaCl solution, suspended by vortexing, and subsequently plated on selective and differential agar medium plates.

The viable bacterial cells in feces were inoculated on the following selective media: Columbia blood agar (total bacteria count; Becton Dickinson, Heidelberg, Germany), Chromid CPS agar (*Escherichia coli*, *Proteus*, *Enterococcus* and *Klebsiella*, *Enterobacter*, *Serratia* and *Citrobacter;* BioMerieux, Durham, USA), Rogosa TMB HPR agar (*Lactobacillus*; Heipha, Eppelheim, Germany), *Bifidobacterium* agar (*Bifidobacteria*; Becton Dickinson, Germany), Schaedler agar (*Bacteroides*; Heipha, Eppelheim, Germany), and SPM agar (*Clostridium* spp.; Heipha, Eppelheim, Germany). The plates were incubated under either aerobic or anaerobic conditions at 37 °C for 24 and 48 h.

Both cultures and microscopic observations determined the presence of fecal fungi. Samples were suspended in 0.85% sterile NaCl solution containing trypsin and antibiotics (penicillin–streptomycin). Afterward, samples were inoculated into two Sabouraud agar plates with antibiotics (gentamicin and chloramphenicol). After 2–5 days, yeast colonies were transferred to differential plates were assigned to the taxonomy species group. Molds were examined by morphological observation after 5–7 days of incubation.

To analyze anaerobic, unculturable bacteria such as *Akkermansia muciniphila* and *Faecalibacterium prausnitzii* and determine *Clostridium difficile* numbers, quantitative polymerase chain reaction was used.

#### DNA isolation and quantitative PCR analysis

Bacterial DNA from stool samples was extracted using RIDA Xtract kit in accordance with the manufacturer's instructions. To estimate bacterial quantity, qPCR was performed with the use of RIDA GENE *Akkermansia muciniphila*, RIDA GENE *Faecalibacterium prausnitzii,* and RIDA GENE *Clostridium difficile* kits in accordance with the manufacturer's instructions (R-Biopharm AG, Darmstadt, Germany). The thermal profile was as follows: initial denaturation (1 min, 95 °C), then 45 cycles of denaturation (15 s, 95 °C) and annealing/extension (30 s, 60 °C). The total reaction mixture volume was 25 μL containing 19.9 μL reaction mix, 0.1 μL Taq Polymerase, and 5 μL DNA-extract. The standard curve was generated with standard DNA A: 5 × 10^2^ copies/reaction, standard DNA B: 5 × 10^4^ copies/reaction, and standard DNA C: 5 × 10^6^ copies/reaction. The reaction was performed in the RotorGene thermal cycler (QIAGEN, Manheim, Germany). The final number of bacteria/gram of stool was obtained by multiplying by 200 due to the dilution factor of the stool sample during extraction.

### Statistical analyses

Several statistical analyses were performed using Statistica ver. 13 software (TIBCO Software, Tulsa, USA). The distributions of the continuous variables were assessed with the Shapiro–Wilk test. As the data was not normally distributed, a nonparametric, 2-sided Kruskal–Wallis test with Dunn's *post-hoc* test for multiple comparisons was used. Nonparametric Spearman's rank correlation test was applied to determine the strength of a link between microbe species. For individual comparisons, a *p*-value of < 0.05 was considered significant. The results were described using nonparametric descriptive statistics. The correlation matrix for factor analysis of principal component loadings for multi-presence, multiple-bacteria strains was computed to determine homogenous subgroups. It was calculated for 12 strains with unrotated factor rotation. Missing data were case wise deleted, 80 cases were processed, and 73 valid cases were accepted. Based on the multi-presence of microorganisms, using Ward's method cluster joining and Euclidean distances, the bacteria genera were clustered.

### Institutional review board statement

The study was conducted according to the guidelines of the Declaration of Helsinki, and approved by the Institutional Review Board of Poznan University of Medical Sciences. However, it concluded that it is not a clinical trial and does not have the characteristics of a medical experiment (date of approval: 08/12/2020). According to the regulations of local IRB our study requires fully anonymized data of the participants.

### Informed consent statement

Informed consent was obtained from all parents or legally authorized representatives, and identifying information was removed from each sample.

## Supplementary Information

Supplementary Tables.
