# Supplementary material for: Commercial microbiota test revealed differences in the composition of intestinal microorganisms between children with autism spectrum disorders and neurotypical peers
Source: Sci Rep. 2021 Dec 20;11:24274. doi: 10.1038/s41598-021-03794-8 (PMC8688445; doi:10.1038/s41598-021-03794-8)
Supplement: Supplementary file 1 — Supplementary Tables. [file 41598_2021_3794_MOESM1_ESM.docx]

# Supplementary data

**Commercial microbiota test revealed differences in the composition of intestinal microorganisms between children with autism spectrum disorders and neurotypical peers**

**Magdalena Jendraszak**^1,#^ (ORCID ID: 0000-0001-8139-9532), **Mirosława Gałęcka**^2,#^ (ORCID ID: 0000-0003-0801-7985), **Małgorzata Kotwicka**^1^ (ORCID ID: 0000-0002-9802-374X), **Aleksandra Regdos**^2^ (ORCID ID: 0000-0003-1158-0904), **Michalina Pazgrat-Patan**^2^ (ORCID ID: 0000-0002-4584-1036), **Mirosław Andrusiewicz**^1,*^ (ORCID ID: 0000-0002-8781-3447)

^1^ Chair and Department of Cell Biology, Faculty of Health Sciences, Poznan University of Medical Sciences, Rokietnicka 5D, 60-806 Poznań, Poland

^2^ Institute of Microecology, Sielska 6, 60-129 Poznań, Poland.

^#^Magdalena Jendraszak and Mirosława Gałęcka contributed equally to this work

^*^**Corresponding author**

Mirosław Andrusiewicz

E-mail: [andrus@ump.edu.pl](mailto:andrus@ump.edu.pl), Phone: +4861854 7166, Fax: +4861854 7169

Table S1. Summary of the various species of microorganisms found in stool from the participants

| Microorganisms/group | N | Me | Min | Max | Q1 | Q3 |
| --- | --- | --- | --- | --- | --- | --- |
| **Enterobacteriaceae** |  |  |  |  |  |  |
| *Escherichia coli* | 73 | 4E+06 | 2E+03 | 6E+08 | 9E+05 | 2E+07 |
| Non-Lactose Fermenting *E. coli* | 73 | 2E+03 | 2E+03 | 2E+07 | 2E+03 | 2E+03 |
| *Proteus spp.* | 73 | 2E+03 | 2E+03 | 2E+03 | 2E+03 | 2E+03 |
| *Providencia spp.* | 73 | 2E+03 | 2E+03 | 2E+03 | 2E+03 | 2E+03 |
| *Morganella spp* | 73 | 2E+03 | 2E+03 | 1E+07 | 2E+03 | 2E+03 |
| *Pseudomonas spp* | 73 | 2E+03 | 2E+03 | 4E+04 | 2E+03 | 2E+03 |
| *Klebsiella spp* | 73 | 2E+03 | 2E+03 | 6E+06 | 2E+03 | 2E+03 |
| *Enterobacter spp.* | 73 | 2E+03 | 2E+03 | 9E+06 | 2E+03 | 2E+03 |
| *Citrobacter Spp* | 73 | 2E+03 | 2E+03 | 3E+08 | 2E+03 | 2E+03 |
| *Serratia Spp* | 73 | 2E+03 | 2E+03 | 2E+06 | 2E+03 | 2E+03 |
| *Hafnia alvei* | 73 | 2E+03 | 2E+03 | 4E+04 | 2E+03 | 2E+03 |
| **Probiotics bacteria** |  |  |  |  |  |  |
| *Bifidobacterium spp.* | 73 | 7E+08 | 7E+05 | 7E+09 | 2E+07 | 2E+09 |
| *Lactobacillus spp.* | 73 | 2E+04 | 2E+03 | 1E+07 | 2E+03 | 4E+05 |
| ***Clostridium spp*** | 73 | 2E+03 | 2E+03 | 4E+06 | 2E+03 | 4E+04 |
| ***Bacteroides spp.*** | 73 | 2E+09 | 2E+07 | 5E+09 | 1E+09 | 3E+09 |
| ***Faecalibacterium prausnitzii*** | 73 | 3E+07 | 3E+05 | 2E+09 | 4E+06 | 3E+08 |
| ***Akkermansia muciniphila*** | 73 | 2E+05 | 1E+03 | 3E+08 | 6E+03 | 4E+06 |
| ***Enterococcus spp.*** | 73 | 3E+04 | 2E+03 | 1E+08 | 2E+03 | 1E+06 |
| ***Candida albicans*** | 73 | 1E+02 | 1E+02 | 2E+05 | 1E+02 | 1E+03 |

N – number of cases; Me – median; Min – minimal; Max – Maximal; Q1 – lower quartile; Q3 – upper quartile

Table S2. Summary of the various species of microorganisms found in stool from ASD, ALG and healthy

| Microorganisms/group | N | Me | Min | Max | Q1 | Q3 | | N | Me | Min | Max | Q1 | Q3 | | N | Me | Min | Max | Q1 | Q3 | |
| --- | --- | --- | --- | --- | --- | --- | --- | --- | --- | --- | --- | --- | --- | --- | --- | --- | --- | --- | --- | --- | --- |
|  | Healthy | | | | | | ASD | | | | | | | ALG | | | | | | |  |
| **Enterobacteriaceae** |  |  |  |  |  |  | |  |  |  |  |  |  | |  |  |  |  |  |  | |
| *Escherichia coli* | 16 | 4E+06 | 2E+05 | 6E+07 | 1E+06 | 8E+06 | | 33 | 5E+06 | 2E+03 | 3E+08 | 2E+06 | 1E+07 | | 24 | 2E+06 | 2E+03 | 6E+08 | 5E+05 | 4E+07 | |
| Non-Lactose Fermenting *E. coli* | 16 | 2E+03 | 2E+03 | 2E+06 | 2E+03 | 2E+03 | | 33 | 2E+03 | 2E+03 | 2E+07 | 2E+03 | 2E+03 | | 24 | 2E+03 | 2E+03 | 4E+06 | 2E+03 | 2E+03 | |
| *Proteus spp.* | 16 | 2E+03 | 2E+03 | 2E+03 | 2E+03 | 2E+03 | | 33 | 2E+03 | 2E+03 | 2E+03 | 2E+03 | 2E+03 | | 24 | 2E+03 | 2E+03 | 2E+03 | 2E+03 | 2E+03 | |
| *Providencia spp.* | 16 | 2E+03 | 2E+03 | 2E+03 | 2E+03 | 2E+03 | | 33 | 2E+03 | 2E+03 | 2E+03 | 2E+03 | 2E+03 | | 24 | 2E+03 | 2E+03 | 2E+03 | 2E+03 | 2E+03 | |
| *Morganella spp* | 16 | 2E+03 | 2E+03 | 2E+03 | 2E+03 | 2E+03 | | 33 | 2E+03 | 2E+03 | 1E+07 | 2E+03 | 2E+03 | | 24 | 2E+03 | 2E+03 | 2E+03 | 2E+03 | 2E+03 | |
| *Pseudomonas spp* | 16 | 2E+03 | 2E+03 | 2E+04 | 2E+03 | 2E+03 | | 33 | 2E+03 | 2E+03 | 4E+04 | 2E+03 | 2E+03 | | 24 | 2E+03 | 2E+03 | 2E+03 | 2E+03 | 2E+03 | |
| *Klebsiella spp* | 16 | 1E+04 | 2E+03 | 2E+06 | 2E+03 | 8E+04 | | 33 | 2E+03 | 2E+03 | 2E+06 | 2E+03 | 2E+03 | | 24 | 2E+03 | 2E+03 | 6E+06 | 2E+03 | 2E+03 | |
| *Enterobacter spp.* | 16 | 2E+03 | 2E+03 | 9E+06 | 2E+03 | 2E+03 | | 33 | 2E+03 | 2E+03 | 1E+06 | 2E+03 | 2E+03 | | 24 | 2E+03 | 2E+03 | 3E+05 | 2E+03 | 2E+03 | |
| *Citrobacter Spp* | 16 | 2E+03 | 2E+03 | 3E+06 | 2E+03 | 2E+03 | | 33 | 2E+03 | 2E+03 | 4E+06 | 2E+03 | 2E+03 | | 24 | 2E+03 | 2E+03 | 3E+08 | 2E+03 | 2E+03 | |
| *Serratia Spp* | 16 | 2E+03 | 2E+03 | 2E+03 | 2E+03 | 2E+03 | | 33 | 2E+03 | 2E+03 | 2E+06 | 2E+03 | 2E+03 | | 24 | 2E+03 | 2E+03 | 2E+03 | 2E+03 | 2E+03 | |
| *Hafnia alvei* | 16 | 2E+03 | 2E+03 | 4E+04 | 2E+03 | 2E+03 | | 33 | 2E+03 | 2E+03 | 2E+04 | 2E+03 | 2E+03 | | 24 | 2E+03 | 2E+03 | 2E+03 | 2E+03 | 2E+03 | |
| ***Probiotics bacteria*** |  |  |  |  |  |  | |  |  |  |  |  |  | |  |  |  |  |  |  | |
| *Bifidobacterium spp.* | 16 | 9E+08 | 7E+05 | 5E+09 | 5E+08 | 2E+09 | | 33 | 4E+08 | 7E+05 | 4E+09 | 1E+07 | 1E+09 | | 24 | 6E+08 | 1E+06 | 7E+09 | 6E+07 | 2E+09 | |
| *Lactobacillus spp.* | 16 | 7E+04 | 2E+03 | 1E+07 | 2E+03 | 4E+05 | | 33 | 2E+04 | 2E+03 | 6E+06 | 2E+03 | 8E+05 | | 24 | 2E+03 | 2E+03 | 4E+06 | 2E+03 | 5E+04 | |
| ***Clostridium spp*** | 16 | 2E+03 | 2E+03 | 4E+06 | 2E+03 | 2E+03 | | 33 | 2E+03 | 2E+03 | 2E+06 | 2E+03 | 4E+04 | | 24 | 2E+03 | 2E+03 | 5E+05 | 2E+03 | 4E+04 | |
| ***Bacteroides spp.*** | 16 | 2E+09 | 9E+08 | 5E+09 | 2E+09 | 3E+09 | | 33 | 2E+09 | 2E+07 | 5E+09 | 1E+09 | 3E+09 | | 24 | 1E+09 | 2E+07 | 4E+09 | 7E+08 | 2E+09 | |
| ***Faecalibacterium prausnitzii*** | 16 | 2E+07 | 2E+06 | 5E+08 | 6E+06 | 5E+07 | | 33 | 6E+07 | 3E+05 | 2E+09 | 3E+06 | 3E+08 | | 24 | 3E+07 | 6E+05 | 9E+08 | 3E+06 | 4E+08 | |
| ***Akkermansia muciniphila*** | 16 | 5E+04 | 1E+03 | 3E+06 | 1E+03 | 4E+05 | | 33 | 3E+05 | 1E+03 | 1E+08 | 2E+04 | 1E+07 | | 24 | 7E+04 | 1E+03 | 3E+07 | 7E+03 | 4E+06 | |
| ***Enterococcus spp.*** | 16 | 2E+03 | 2E+03 | 1E+07 | 2E+03 | 2E+06 | | 33 | 8E+04 | 2E+03 | 1E+08 | 2E+03 | 8E+05 | | 24 | 6E+04 | 2E+03 | 8E+06 | 2E+03 | 2E+06 | |
| ***Candida albicans*** | 16 | 1E+02 | 1E+02 | 1E+05 | 1E+02 | 5E+03 | | 33 | 5E+02 | 1E+02 | 5E+04 | 1E+02 | 2E+03 | | 24 | 1E+02 | 1E+02 | 2E+05 | 1E+02 | 8E+02 | |

ALG – Allergies; ASD – Autism spectrum disorders; N – number of cases; Me – median; Min – minimal; Max – Maximal; Q1 – lower quartile; Q3 – upper quartile

Table S3. Summary of the various species of microorganisms found in stool from ASD, ALG and healthy divided by probiotics usage.

| Microorganisms/group | N | Me | Min | Max | Q1 | Q3 | N | Me | Min | Max | Q1 | Q3 | N | Me | Min | Max | Q1 | Q3 |
| --- | --- | --- | --- | --- | --- | --- | --- | --- | --- | --- | --- | --- | --- | --- | --- | --- | --- | --- |
|  | Healthy | | | | | | ASD | | | | | | ALG | | | | | |
| **Probiotics - Yes** | | | | | | | | | | | | | | | | | | |
| **Enterobacteriaceae** | | | | | | | | | | | | | | | | | | |
| *Escherichia coli* | 9 | 2E+06 | 3E+05 | 6E+07 | 2E+06 | 6E+06 | 12 | 5E+06 | 2E+03 | 3E+08 | 2E+06 | 6E+06 | 7 | 9E+05 | 4E+04 | 8E+07 | 4E+05 | 6E+06 |
| Non-Lactose Fermenting *E. coli* | 9 | 2E+03 | 2E+03 | 2E+03 | 2E+03 | 2E+03 | 12 | 2E+03 | 2E+03 | 1E+06 | 2E+03 | 2E+03 | 7 | 2E+03 | 2E+03 | 2E+05 | 2E+03 | 2E+03 |
| *Proteus spp.* | 9 | 2E+03 | 2E+03 | 2E+03 | 2E+03 | 2E+03 | 12 | 2E+03 | 2E+03 | 2E+03 | 2E+03 | 2E+03 | 7 | 2E+03 | 2E+03 | 2E+03 | 2E+03 | 2E+03 |
| *Providencia spp.* | 9 | 2E+03 | 2E+03 | 2E+03 | 2E+03 | 2E+03 | 12 | 2E+03 | 2E+03 | 2E+03 | 2E+03 | 2E+03 | 7 | 2E+03 | 2E+03 | 2E+03 | 2E+03 | 2E+03 |
| *Morganella spp* | 9 | 2E+03 | 2E+03 | 2E+03 | 2E+03 | 2E+03 | 12 | 2E+03 | 2E+03 | 2E+03 | 2E+03 | 2E+03 | 7 | 2E+03 | 2E+03 | 2E+03 | 2E+03 | 2E+03 |
| *Pseudomonas spp* | 9 | 2E+03 | 2E+03 | 2E+03 | 2E+03 | 2E+03 | 12 | 2E+03 | 2E+03 | 4E+04 | 2E+03 | 2E+03 | 7 | 2E+03 | 2E+03 | 2E+03 | 2E+03 | 2E+03 |
| *Kleibsiella spp* | 9 | 2E+03 | 2E+03 | 1E+05 | 2E+03 | 8E+04 | 12 | 2E+03 | 2E+03 | 2E+06 | 2E+03 | 2E+03 | 7 | 2E+03 | 2E+03 | 6E+06 | 2E+03 | 2E+03 |
| *Enterobacter spp.* | 9 | 2E+03 | 2E+03 | 2E+03 | 2E+03 | 2E+03 | 12 | 2E+03 | 2E+03 | 3E+05 | 2E+03 | 2E+03 | 7 | 2E+03 | 2E+03 | 2E+05 | 2E+03 | 2E+04 |
| *Citrobacter Spp* | 9 | 2E+03 | 2E+03 | 3E+06 | 2E+03 | 2E+03 | 12 | 2E+03 | 2E+03 | 2E+03 | 2E+03 | 2E+03 | 7 | 2E+03 | 2E+03 | 8E+06 | 2E+03 | 2E+03 |
| *Serratia Spp* | 9 | 2E+03 | 2E+03 | 2E+03 | 2E+03 | 2E+03 | 12 | 2E+03 | 2E+03 | 2E+03 | 2E+03 | 2E+03 | 7 | 2E+03 | 2E+03 | 2E+03 | 2E+03 | 2E+03 |
| *Hafnia alvei* | 9 | 2E+03 | 2E+03 | 2E+03 | 2E+03 | 2E+03 | 12 | 2E+03 | 2E+03 | 2E+03 | 2E+03 | 2E+03 | 7 | 2E+03 | 2E+03 | 2E+03 | 2E+03 | 2E+03 |
| **Probiotics bacteria** |  |  |  |  |  |  |  |  |  |  |  |  |  |  |  |  |  |  |
| *Bifidobacterium spp.* | 9 | 9E+08 | 2E+06 | 5E+09 | 7E+08 | 1E+09 | 12 | 9E+08 | 7E+05 | 4E+09 | 2E+07 | 3E+09 | 7 | 6E+07 | 1E+06 | 2E+09 | 3E+06 | 7E+08 |
| *Lactobacillus spp.* | 9 | 1E+05 | 2E+03 | 1E+07 | 2E+04 | 1E+06 | 12 | 1E+05 | 2E+03 | 5E+06 | 2E+03 | 1E+06 | 7 | 2E+03 | 2E+03 | 2E+06 | 2E+03 | 6E+04 |
| ***Clostridium spp*** | 9 | 2E+03 | 2E+03 | 4E+06 | 2E+03 | 2E+03 | 12 | 2E+03 | 2E+03 | 3E+05 | 2E+03 | 2E+03 | 7 | 2E+03 | 2E+03 | 2E+03 | 2E+03 | 2E+03 |
| ***Bacteroides spp.*** | 9 | 2E+09 | 1E+09 | 3E+09 | 2E+09 | 3E+09 | 12 | 2E+09 | 2E+07 | 4E+09 | 1E+09 | 3E+09 | 7 | 1E+09 | 8E+07 | 3E+09 | 4E+08 | 1E+09 |
| ***Faecalibacterium prausnitzii*** | 9 | 3E+07 | 2E+06 | 5E+08 | 6E+06 | 3E+08 | 12 | 6E+07 | 3E+05 | 1E+09 | 3E+06 | 4E+08 | 7 | 1E+07 | 1E+06 | 9E+08 | 2E+06 | 6E+08 |
| ***Akkermansia muciniphila*** | 9 | 7E+04 | 1E+03 | 3E+06 | 1E+03 | 4E+05 | 12 | 2E+05 | 1E+03 | 6E+07 | 4E+03 | 5E+06 | 7 | 2E+04 | 1E+03 | 1E+07 | 1E+03 | 1E+06 |
| ***Enterococcus spp.*** | 9 | 5E+05 | 2E+03 | 1E+07 | 2E+03 | 2E+06 | 12 | 3E+04 | 2E+03 | 1E+07 | 2E+03 | 1E+06 | 7 | 2E+04 | 2E+03 | 2E+06 | 2E+03 | 4E+05 |
| ***Candida albicans*** | 9 | 1E+02 | 1E+02 | 1E+04 | 1E+02 | 5E+02 | 12 | 1E+02 | 1E+02 | 1E+04 | 1E+02 | 5E+03 | 7 | 5E+02 | 1E+02 | 4E+03 | 1E+02 | 1E+03 |
| **Probiotics - No** | | | | | | | | | | | | | | | | | | |
| **Enterobacteriaceae** | | | | | | | | | | | | | | | | | | |
| *Escherichia coli* | 7 | 4E+06 | 2E+05 | 4E+07 | 3E+05 | 4E+07 | 21 | 6E+06 | 2E+03 | 3E+08 | 9E+05 | 2E+07 | 17 | 6E+06 | 2E+03 | 6E+08 | 1E+06 | 6E+07 |
| Non-Lactose Fermenting *E. coli* | 7 | 2E+03 | 2E+03 | 2E+06 | 2E+03 | 2E+03 | 21 | 2E+03 | 2E+03 | 2E+07 | 2E+03 | 2E+03 | 17 | 2E+03 | 2E+03 | 4E+06 | 2E+03 | 2E+03 |
| *Proteus spp.* | 7 | 2E+03 | 2E+03 | 2E+03 | 2E+03 | 2E+03 | 21 | 2E+03 | 2E+03 | 2E+03 | 2E+03 | 2E+03 | 17 | 2E+03 | 2E+03 | 2E+03 | 2E+03 | 2E+03 |
| *Providencia spp.* | 7 | 2E+03 | 2E+03 | 2E+03 | 2E+03 | 2E+03 | 21 | 2E+03 | 2E+03 | 2E+03 | 2E+03 | 2E+03 | 17 | 2E+03 | 2E+03 | 2E+03 | 2E+03 | 2E+03 |
| *Morganella spp* | 7 | 2E+03 | 2E+03 | 2E+03 | 2E+03 | 2E+03 | 21 | 2E+03 | 2E+03 | 1E+07 | 2E+03 | 2E+03 | 17 | 2E+03 | 2E+03 | 2E+03 | 2E+03 | 2E+03 |
| *Pseudomonas spp* | 7 | 2E+03 | 2E+03 | 2E+04 | 2E+03 | 2E+03 | 21 | 2E+03 | 2E+03 | 2E+03 | 2E+03 | 2E+03 | 17 | 2E+03 | 2E+03 | 2E+03 | 2E+03 | 2E+03 |
| *Kleibsiella spp* | 7 | 2E+04 | 2E+03 | 2E+06 | 2E+03 | 2E+05 | 21 | 2E+03 | 2E+03 | 2E+06 | 2E+03 | 2E+03 | 17 | 2E+03 | 2E+03 | 2E+03 | 2E+03 | 2E+03 |
| *Enterobacter spp.* | 7 | 2E+03 | 2E+03 | 9E+06 | 2E+03 | 2E+03 | 21 | 2E+03 | 2E+03 | 1E+06 | 2E+03 | 2E+03 | 17 | 2E+03 | 2E+03 | 3E+05 | 2E+03 | 2E+03 |
| *Citrobacter Spp* | 7 | 2E+03 | 2E+03 | 2E+05 | 2E+03 | 2E+03 | 21 | 2E+03 | 2E+03 | 4E+06 | 2E+03 | 2E+03 | 17 | 2E+03 | 2E+03 | 3E+08 | 2E+03 | 2E+03 |
| *Serratia Spp* | 7 | 2E+03 | 2E+03 | 2E+03 | 2E+03 | 2E+03 | 21 | 2E+03 | 2E+03 | 2E+06 | 2E+03 | 2E+03 | 17 | 2E+03 | 2E+03 | 2E+03 | 2E+03 | 2E+03 |
| *Hafnia alvei* | 7 | 2E+03 | 2E+03 | 4E+04 | 2E+03 | 2E+03 | 21 | 2E+03 | 2E+03 | 2E+04 | 2E+03 | 2E+03 | 17 | 2E+03 | 2E+03 | 2E+03 | 2E+03 | 2E+03 |
| **Probiotics bacteria** |  |  |  |  |  |  |  |  |  |  |  |  |  |  |  |  |  |  |
| *Bifidobacterium spp.* | 7 | 1.E+09 | 7.E+05 | 3.E+09 | 3.E+08 | 3.E+09 | 21 | 4.E+07 | 2.E+06 | 2.E+09 | 1.E+07 | 8.E+08 | 17 | 1.E+09 | 6.E+06 | 7.E+09 | 4.E+08 | 3.E+09 |
| *Lactobacillus spp.* | 7 | 2.E+03 | 2.E+03 | 4.E+05 | 2.E+03 | 3.E+05 | 21 | 2.E+03 | 2.E+03 | 6.E+06 | 2.E+03 | 4.E+05 | 17 | 2.E+03 | 2.E+03 | 4.E+06 | 2.E+03 | 4.E+04 |
| ***Clostridium spp*** | 7 | 2.E+03 | 2.E+03 | 8.E+04 | 2.E+03 | 2.E+04 | 21 | 2.E+03 | 2.E+03 | 2.E+06 | 2.E+03 | 1.E+05 | 17 | 2.E+03 | 2.E+03 | 5.E+05 | 2.E+03 | 4.E+04 |
| ***Bacteroides spp.*** | 7 | 2.E+09 | 9.E+08 | 5.E+09 | 1.E+09 | 4.E+09 | 21 | 2.E+09 | 2.E+08 | 5.E+09 | 1.E+09 | 3.E+09 | 17 | 2.E+09 | 2.E+07 | 4.E+09 | 1.E+09 | 2.E+09 |
| ***Faecalibacterium prausnitzii*** | 7 | 9.E+06 | 2.E+06 | 7.E+07 | 3.E+06 | 3.E+07 | 21 | 6.E+07 | 3.E+05 | 2.E+09 | 4.E+06 | 2.E+08 | 17 | 4.E+07 | 6.E+05 | 8.E+08 | 3.E+06 | 2.E+08 |
| ***Akkermansia mucniphila*** | 7 | 3.E+04 | 1.E+03 | 5.E+05 | 1.E+03 | 3.E+05 | 21 | 3.E+05 | 1.E+03 | 1.E+08 | 4.E+04 | 1.E+07 | 17 | 1.E+05 | 1.E+03 | 3.E+07 | 1.E+04 | 4.E+06 |
| ***Enterococcus spp.*** | 7 | 2.E+03 | 2.E+03 | 2.E+06 | 2.E+03 | 2.E+05 | 21 | 3.E+05 | 2.E+03 | 1.E+08 | 2.E+03 | 8.E+05 | 17 | 1.E+05 | 2.E+03 | 8.E+06 | 2.E+03 | 2.E+06 |
| ***Candida albicans*** | 7 | 1.E+03 | 1.E+02 | 1.E+05 | 1.E+02 | 2.E+04 | 21 | 5.E+02 | 1.E+02 | 5.E+04 | 1.E+02 | 2.E+03 | 17 | 1.E+02 | 1.E+02 | 2.E+05 | 1.E+02 | 5.E+02 |

ALG – Allergies; ASD – Autism spectrum disorders; N – number of cases; Me – median; Min – minimal; Max – Maximal; Q1 – lower quartile; Q3 – upper quartile
